# Maintenance of Periodontally Compromised Teeth Using Periodontal Splints

**DOI:** 10.1155/ijod/7119673

**Published:** 2025-08-30

**Authors:** Janina Golob Deeb, Caroline K. Carrico, Anne Miller, Jacqueline Bennett, Amirreza Ghassemi

**Affiliations:** ^1^Department of Periodontics, Virginia Commonwealth University, Richmond, Virginia, USA; ^2^Department of Dental Public Health and Policy, Virginia Commonwealth University, Richmond, Virginia, USA; ^3^Art of Dentistry, Tucson, Arizona, USA

**Keywords:** provisional prosthesis, splint, tooth mobility

## Abstract

**Objective:** Periodontal splints offer a noninvasive and inexpensive treatment modality to stabilize mobile teeth. This study evaluated periodontal splints to examine their longevity and long-term stability.

**Methods:** A retrospective chart review was performed for patients who received splints on mandibular or maxillary anterior teeth. Data collected included patient age, gender, indication for splint placement, date of placement, teeth involved, percent bone loss, presence of a pontic, and type of reinforcement. Data were summarized with descriptive statistics. Differences in bone loss were analyzed using *t*-tests.

**Results:** A total of 154 cases were included in the study. The average patient age was 57.2 and ranged from 17 to 93, with nearly equal rates of males and females (54% vs. 46%). Procedures were performed from 2003 until 2024. The majority were mandibular splints with (20%) or without a pontic (47%). Maxillary splints accounted for 33% of the sample (12% with pontic and 21% without). The nature of the splint was periodontal for 68% (*n* = 105) of the cases. Trauma accounted for 18% and included incidents with oral piercings and avulsion. Periodontal cases were assessed for bone loss where data were available. The average loss was 76.5% and ranged from 30%to 100%. Bone loss was not significantly associated with the location of the splint (*p*-value = 0.3690), patient gender (*p*-value = 0.3391), or use of splint reinforcement (*p*-value = 0.3548). Only 40% of splints were reinforced. More than half of the cases had a longevity of 10 or more years (*n* = 82, 55%).

**Conclusion:** Splinting can provide long-term support to mobile teeth and aid in maintaining periodontally compromised teeth. In replacing single anterior teeth, a splint with a pontic can provide a stable provisional prosthesis as an alternative to a temporary removable appliance.

**Summary**

- Splinting can provide long-term support to mobile teeth and aid in maintaining periodontally compromised teeth.
- When used with a pontic, it can provide a stable provisional prosthesis to replace single anterior tooth.

## 1. Introduction

Splinting is a technique in dentistry utilized to stabilize teeth with mobility. Patients experience tooth mobility for various reasons, most frequently encountered among them being periodontal disease, trauma, or orthodontic treatment [1, 2].

Tooth mobility in patients with periodontitis can lead to discomfort when chewing and speaking and may be caused by inflammation, loss of clinical attachment, or functional and parafunctional forces on teeth [3, 4]. The goal of splinting is to stabilize mobile teeth by evenly distributing occlusal forces hence, improving comfort, function, esthetics, and oral hygiene [1, 3]. Splinting teeth allows the distribution of forces from mobile teeth to their neighboring teeth to gain support from stronger adjacent teeth and to prolong the life expectancy and function of teeth with mobility. More stable teeth provide the patient with improved comfort, self-image, function, and esthetics [5]. A number of different removable and fixed techniques have been described for splinting teeth. The most frequently applied fixed splints for definitive incisor splinting are fabricated from composite resin with or without reinforcement fibers [3]. Materials used for splinting have evolved over time and currently, most splints are fabricated using tooth color-matched composite resin [3]. Reinforcement with wires or bondable fibers, like polyethylene mesh, may further stabilize splinted mobile teeth and prevent injury to the periodontal ligament [2, 6]. Some of the disadvantages of reinforced splints include more difficult access for oral hygiene practices [7], and delamination or fractures of composite resin material over time [6], requiring often more frequent maintenance appointments [8, 9]. Digital techniques to fabricate splints using CAD-CAM technology to stabilize mobile mandibular anterior teeth utilize metal alloy materials [10], or aesthetically superior polyetherketoneketone (PEKK), a high-performance thermoplastic resin [11], overcoming some of the challenges described with direct splint fabrication.

Periodontally compromised splinted teeth can be maintained over a long period of time. Therefore, splints supporting these teeth should be stable, cleansable, esthetically pleasing, safe, easily fabricated, repairable, and should not adversely affect oral and periodontal health. Periodontal splints are primarily utilized for the temporary stabilization of teeth. However, due to their longevity beyond the initial temporary objective, splints in certain cases may serve as a predictable and inexpensive treatment option for patients who wish to maintain mobile teeth or are not candidates for dental implants or other prosthetic treatment options due to cost or medical conditions. Alternative treatment options such as fixed and removable partial dentures may not be suitable for certain cases as they require preparation of adjacent teeth, devices that are removable, and can increase torque on teeth, further worsening tooth mobility [6]. Splinting compromised teeth may avoid tooth extraction and delay more extensive prosthodontic treatment [7].

Based on the application, splints are divided into extracoronal (EC) and intracoronal (IC) splints. EC splints are applied superficially on tooth surface relying on strong adhesive bond between resin material and enamel, while IC splints require preparation of the channel inside the coronal structure of the tooth to accommodate fibers that provide reinforcement. Both EC and IC splints can be reinforced with bondable fibers, wires, or meshes, which add complexity to the technique and bulkiness to the splint. Reinforced splints are more plaque retentive, less esthetic due to bulkiness and embedded reinforcements, and more difficult to repair.

Splinting can also be employed as a provisional prosthesis to replace a missing anterior tooth. Loss of an anterior tooth can be a traumatic experience for a patient, and may be accompanied by adjacent tooth mobility [6]. Although dental implants are usually the first choice for the treatment of missing anterior teeth, some patients may have unfavorable biological environment for an implant or cannot immediately afford the expense of treatment [6, 8]. Provisional prosthesis provides an inexpensive esthetic treatment option with immediate chair-site implementation. Provisional prosthesis can make use of the coronal part of the extracted natural tooth as a pontic, and serve as a temporary esthetic tooth replacement option [2].

Periodontal splints can be considered as either temporary, provisional, or permanent. Temporary splints are meant to be worn for less than 6 months, and provisional prosthesis may be in service for a few months to a few years with an established endpoint of splint therapy [4]. Splints may be used for long-term tooth support as the 3-year survival rate of approximately 74% before fracture or debonding has been reported for splints of mandibular anterior teeth [3]. Limited current evidence exists on the longevity, stability, and the maintenance of splints and splinted teeth to understand their long-term use in clinical practice [4].

The goal of this study was to analyze the cases that utilized splinting therapy, including demographic and teeth characteristics of patients receiving this treatment modality. The specific aims of this research were to determine the teeth that are most frequently splinted, identify the etiologic factors leading to tooth mobility (periodontal disease, dental trauma, missing tooth, and orthodontic treatment), and identify demographic components of these patients (age and percent bone loss).

## 2. Materials and Methods

This retrospective study was approved by the Institutional Review Board at Virginia Commonwealth University (IRB HM20027224). Charts reviewed included patients who received IC or EC splints made directly with composite resin material chair-side to stabilize mobile teeth or to replace a missing anterior tooth with a provisional pontic. They were further divided into splints made solely out of resin material or reinforced with bondable fibers embedded in composite material intra- or extracoronally. Data collection included the age and gender of patients, indication for the splint, teeth involved, missing teeth replaced by a provisional prosthesis with a pontic as a part of the splint, percent of bone loss around affected teeth, year of splinting, and status of it at present date if available. Bone loss was assessed on two-dimensional radiographic images, either periapical or panoramic radiographs at the time of splinting. Percentage of bone loss was estimated with reference points of cementoenamel junction and apex of the tooth, excluding 2 mm of supracrestal attachment. Individuals younger than 14 years, those who suffer from complex dental or skeletal disorders were excluded from the study.

Deidentified data were collected retrospectively from patient charts. Data were summarized with descriptive statistics (counts, percentages or mean, and standard deviation [SD]). Differences in bone loss were analyzed using parametric and nonparametric methods, including *t*-tests, ANOVA, Wilcoxon, and Kruskal–Wallis tests. SAS EG v.8.2 (SAS Institute, Cary, NC) was used for all analyses.

Splinting is considered a temporary remedy for stabilizing mobile teeth; however, the majority of patients maintain them well beyond the duration of their intended temporary purpose owing to improved masticatory function and comfort of mobile anterior teeth and acceptable esthetics with contemporary composite splints. Splinted stabilized mobile anterior teeth distribute occlusal forces more evenly, hence improving comfort, function, esthetics, and oral hygiene [1, 3]. Since our experimental data collection was of retrospective nature and subjective reporting on improved chewing function, esthetics, and comfort of mobile anterior teeth was unavailable, our conclusions on their favored serviceability by patients were drawn from their longevity and willingness of patients to maintain the splinted teeth and not seek further treatments to replace splinted teeth considering it a suitable longer-term treatment option. Following splinting, teeth become more stable and provide patient with improved comfort, self-image, function, and esthetics. This prolongs the life expectancy of compromised teeth and provides stability for the periodontium to reattach.

## 3. Results

**Figure 1:**
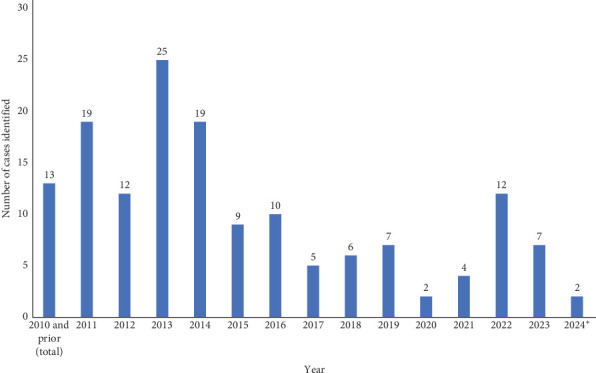
Summary of cases per year. *⁣*^*∗*^Incomplete data available for calendar year 2024.

A total of 154 cases were included in the study (Table 1). The average patient age was 57.2 and ranged from 17 to 93. There were nearly equal rates of males and females (54% vs. 46%). Procedures were performed from 2003 until 2024, with an average number of 10.5 per year for 2011 through 2023 due to incomplete data prior to 2011 and the incomplete calendar year for 2024.  Figure 1 displays the rate of cases identified per year. Incomplete data available for calendar year 2024.

**Figure 2:**
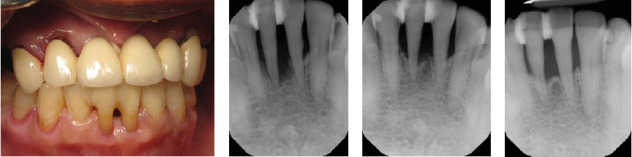
Periodontally compromised mandibular incisors were splinted in 2016 (A and B). Radiographically observed long-term stability in 2019 (C) and 2022 (D).

**Figure 3:**
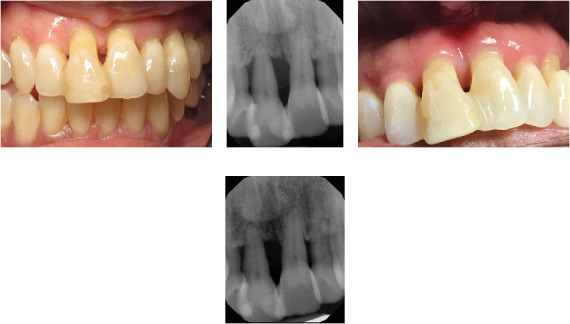
Periodontally compromised maxillary incisors were splinted in 2014 (A and B). Clinically (C) and radiographically (D) observed long-term stability in 2023.

**Figure 4:**
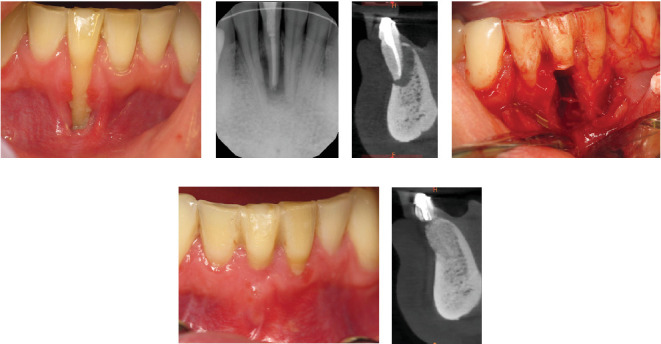
Mandibular incisor with mucogingival defect (A) and periapical periodontitis (B and C) before extraction. Tooth was extracted (D) and crown used as a pontic in provisional prosthesis and splinted to the adjacent teeth. Favorable healing was observed clinically (E) and radiographically at a 2-year follow-up (F).

Figures 2–4 demonstrate sample cases included in the analysis. These cases include periodontally compromised mandibular incisors that were splinted in 2016 (Figure 2A, B) and demonstrated long-term stability radiographically in 2019 (Figure 2C) and 2022 (Figure 2D). Similarly, Figure 3 depicts compromised maxillary incisors that were splinted in 2014 (Figure 3A, B), and demonstrated clinically (Figure 3C) and radiographically (Figure 3D), observed long-term stability in 2023.  Figure 4 demonstrates successful treatment of a mandibular mucogingival defect in which the affected tooth was extracted and the crown portion was splinted to adjacent teeth as a pontic in a provisional prosthesis. Favorable healing is demonstrated clinically and radiographically at a 2-year follow-up (Figure 4E,F).

The majority of cases were mandibular splints (67%) with (20%) or without a pontic (47%). Maxillary splints accounted for the remaining 33% of the cases (12% with and 21% without pontic). The type of the splint was periodontal for 68% (*n* = 105) of the cases. Trauma accounted for 18% of the cases and included incidents with oral piercings and avulsion most commonly. The remaining cases also categorized as “other” included retained deciduous teeth and congenitally missing teeth among others. Less than half of the cases were reinforced splints (*n* = 61, 40%). Reinforcements included: EC splints with embedded fiber (*n* = 30, 49%), IC splints with embedded fiber (*n* = 12, 20%), and other methods (*n* = 19, 31%). More than half of the cases had a longevity of 10 or more years (*n* = 82, 55%). Summary of cases included is provided in Table 1.

Periodontal cases were assessed for bone loss where data were available. There were 62 periodontal cases with nonzero bone loss. The average loss was 76.5% and ranged from 30% to 100%. Bone loss was not significantly associated with location of the splint (*p*-value = 0.3690), patient gender (*p*-value = 0.3391), or use of splint reinforcement (*p*-value = 0.3548). When the type of splint was separated into jaw (*p*-value = 0.3840) and presence of a pontic (*p*-value = 0.3913), the results were similar to the four-level grouping of jaw and presence of pontic. Results are presented in Table 2.

**Figure 5:**
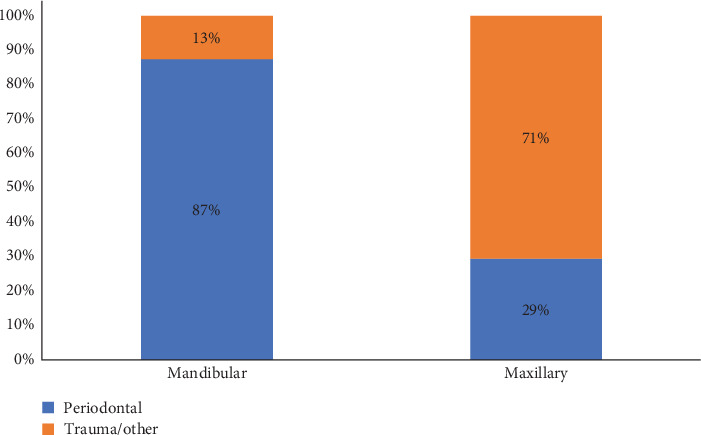
Association between jaw and nature of splint (*p*-value < 0.0001).

As may have been anticipated, maxillary splints were more commonly trauma-related (71%) than periodontal (29%), which was significantly different from mandibular, which were more likely periodontal in nature (87%) than trauma (13%) (*p*-value < 0.0001). Results are provided in Figure 5.

## 4. Discussion

This study analyzed cases with existing splints to determine their long-term stability and service. Research supports the use of tooth stabilization and splinting to improve the prognosis [12]. Teeth are splinted for a variety of reasons. These indications include tooth stabilization for orthodontic retention, repositioning and reimplanting a tooth that has been traumatized, teeth in primary or secondary occlusal trauma, replacing a missing tooth, and overcoming progressive mobility due to periodontal disease, migration, or pain in function [13]. Findings of this study are in accordance with previously reported >observations, observing that periodontally compromised splinted teeth have a high survival rate and stability as a part of supportive periodontal therapy (SPT). The reported survival rate of the splints until fracture or debonding is 74.4% at 3 years [3]. Periodontal pocket depths around splinted teeth have been reported to decrease, and bone levels and clinical attachment level (CAL) remained stable over a 10-year period [3, 6].

Limited evidence exists on the durability and longevity of these splints; however, in daily practice, they outlive expectations and provide great patient satisfaction. Splints have been used in dentistry for several decades; however, their long-term survival faced challenges in the past. Fabricating an esthetically acceptable and long-term functional splint was difficult due to shortcomings of composite materials, chemical material incompatibility of embedded wires, bulkiness, and delamination problems over time. Their esthetic and functional properties have improved steadily with continuously evolving and improving adhesive, strength, and color properties of contemporary composite materials.

Splinting of mobile teeth to relatively healthy and stable adjacent teeth has been used to improve comfort and provide better control of occlusion [7]. It is mainly used for temporary stabilization of teeth during various dental and surgical therapies that result in or are caused by tooth mobility. This treatment may also present a treatment modality for patients who prefer to keep their mobile teeth, cannot afford the expense of the dental implant therapy, or are medically unfit to undergo surgical procedures. Based on survivability noted in this and previous studies, this treatment modality may enable maintenance of periodontally compromised teeth for several years and delay their removal and replacement.

Limitations of this study include missing or minimal information obtained from the records and patient attrition, which affected the inability to document the current state of periodontal splints. Due to retrospective analysis, no surveying of subjective findings from patients' perceptions was available, along with missing clinical indices to depict clinical findings around splinted teeth before and after splinting. These should be tasks addressed in future long-term prospective studies utilizing splinting therapy as treatment modality.

Sample size for this study was determined by the available data. Post hoc power analyses found low statistical power across the analyses in Table 2, at about 15% compared to standard goal of 80%. However, the clinical differences and effect sizes demonstrated in this table also reflect differences in percent of bone loss of less than 5%. While a larger sample size could power these results to be statistically significant, they would, likely, lack clinical relevance.

This research provides an insight into the utilization of splinting as a noninvasive and inexpensive treatment modality and may be used as an alternative treatment to tooth extraction.

## 5. Conclusion

Splinting is a suitable option for improving the masticatory function, esthetics, and comfort of mobile anterior teeth.

## Figures and Tables

**Table 1:** Summary of cases included.

Category	*n*	%
Type of splint	—	—
Mandibular	72	47
Mandibular + pontic	31	20
Maxillary	32	21
Maxillary + pontic	19	12
Nature of splint	—	—
Periodontal	105	68
Trauma	28	18
Other	21	14
Gender	—	—
Male	82	54
Female	71	46
Splint reinforcement		—
Yes	61	40
No	93	60
Type of reinforcement		—
EC Ribbond	30	49
IC Ribbond	12	20
Other	19	31
Time of splint	—	—
<10 years	67	45
10+ years	82	55
Jaw	—	—
Mandibular	103	67
Maxillary	51	33
Pontic	—	—
Yes	104	68
No	50	32

**Table 2:** Bone loss in periodontal cases with available data (*n* = 62).

Category	*n*	Mean	SD	*p*-Value (parametric)	*p*-Value (nonparametric)
Overall	62	76.5	14.0	—	—
Type of splint	—	—	—	0.3690	0.3677
Mandibular	33	78.0	12.2	—	—
Mandibular + pontic	17	75.9	18.4	—	—
Maxillary	10	76.0	10.7	—	—
Maxillary + pontic	2	60.0	14.1	—	—
Gender	—	—	—	0.3391	0.3262
Male	21	79.0	13.7	—	—
Female	40	75.4	14.3	—	—
Splint reinforcement		—	—	0.3548	0.2929
Yes	17	73.8	13.9	—	—
No	45	77.6	14.1	—	—
Jaw	—	—	—	0.3840	0.2321
Mandibular	50	77.3	14.4	—	—
Maxillary	12	73.3	12.3	—	—
Pontic	—	—	—	0.3913	0.7788
Yes	19	74.2	18.4	—	—
No	43	77.6	11.8	—	—

*Note: p*-Value from ANOVA or *t*-test as appropriate; nonparametric *p*-value from Wilcoxon or Kruskal–Wallis test.

## Data Availability

Based on IRB approval criteria, study data are not available for sharing.
